# Yak IGF2 Promotes Fibroblast Proliferation Via Suppression of IGF1R and PI3KCG Expression

**DOI:** 10.3390/genes9030169

**Published:** 2018-03-20

**Authors:** Quanwei Zhang, Qi Wang, Jishang Gong, Jiaxing Du, Yong Zhang, Xingxu Zhao

**Affiliations:** 1 College of Life Science and Technology, Gansu Agriculture University, Lanzhou 730070, China; zhangqw@gsau.edu.cn (Q.Z.); djx813@163.com (J.D.); zhychy@163.com (Y.Z.); 2 College of Veterinary Medicine, Gansu Agriculture University, Lanzhou 730070, China; forever45214125@126.com (Q.W.); gongjishang@126.com (J.G.)

**Keywords:** yak fibroblasts, Insulin-like growth factor 2, Insulin-like growth factor 1 receptor 1, Phosphatidylinositol 3-kinase, catalytic subunit gamma PI3KCG, proliferation

## Abstract

Insulin-like growth factor 2 (IGF2) recapitulates many of the activities of insulin and promotes differentiation of myoblasts and osteoblasts, which likely contribute to genetic variations of growth potential. However, little is known about the functions and signaling properties of IGF2 variants in yaks. The over-expression vector and knockdown sequence of yak *IGF2* were transfected into yak fibroblasts, and the effects were detected by a series of assays. IGF2 expression in yak muscle tissues was significantly lower than that of other tissues. In yak fibroblasts, the up-regulated expression of *IGF2* inhibits expression of IGF1 and insulin-like growth factor 2 receptor (IGF2R) and significantly up-regulates expression of IGF1R. Inhibition of *IGF2* expression caused the up-regulates expression of IGF1, IGF1R and IGF2R. Both over-expression and knockdown of *IGF2* resulted in up-regulation of threonine protein kinase 1 (Akt1) expression and down-regulation of phosphatidylinositol 3-kinase, catalytic subunit gamma (PIK3CG). Cell cycle and cell proliferation assays revealed that over-expression of *IGF2* enhanced the DNA synthesis phase and promoted yak fibroblasts proliferation. Conversely, knockdown of *IGF2* decreased DNA synthesis and inhibited proliferation. These results suggested that *IGF2* was negatively correlated with *IGF1R* and *PIK3CG* and demonstrated an association with the IGFs-PI3K-Akt (IGFs-phosphatidylinositol 3-kinase- threonine protein kinase) pathway in cell proliferation and provided evidence supporting the functional role of *IGF2* for use in improving the production performance of yaks.

## 1. Introduction

The yak (*Bos grunniens*) is a remarkable domestic animal in the northwest areas of China. Limited by environmental factors such as hypoxia, severe cold and strong ultraviolet radiation [1,2], the body mass and size of yaks are much smaller than those of cattle. Moreover, yaks have been grazed for long periods without supplementary feeding [3], which has led to poor nutrition and low reproduction rate [4]. Locally, an underdeveloped economy and outdated scientific technologies have limited the scientific study of yaks compared to other domesticated livestock. Because of the economic and transportation value of yaks, it is important to resolve the shortcomings in reproductive performance. Our previous study suggested insulin-like growth factor 2 (IGF2) is closely related to the growth and development of cattle and yaks [5]. The spatial structure of yak IGF2 is similar to that of human and rat IGF2, but the functional binding sites are significantly different [2]. IGF2 is considered to be a candidate marker for yak breeding that can improve yak production performance. However, little is known about the function and signaling properties of IGF2 variants in livestock, especially in yaks. 

IGFs, including IGF1 and IGF2, are evolutionarily conserved peptides structurally related to insulin (INS) [6], and were discovered to be mediators of growth hormone that affect growth and differentiation of bone and skeletal muscle [7]. The IGFs pathway is a conserved signaling pathway that consists of multiple IGF ligands, IGF receptors, insulin-like growth factor binding proteins (IGFBPs), and the intracellular signal transduction network. IGFs repeat many of the activities of INS and also promote differentiation of myoblast and osteoblast tissues into muscle and bone [7], which likely contribute to genetic variations of growth potential [8]. It has been reported that IGF2 knockout leads to a significant reduction of body size (≈60% of normal) in mice [9]. IGFs are essential for growth and survival by suppressing apoptosis and promoting cell cycle progression, angiogenesis, and metastatic activities in various cancers [10]. Previous studies reported that INS and IGF1 are specific for their cognate receptors, whereas IGF2 has similar affinities to the IGF1 receptor (IGF1R) and the short isoform of the insulin receptor (INSR) [10,11]. The actions of IGF1 and IGF2 are likely mediated through IGF1R that is involved in cell transformation. IGF2R is the most closely related to IGF2 but has no tyrosine kinase and lysosomal degradation activity. The regulatory mechanisms of IGF2 and IGF2R remain unknown. The binding of IGF2 to IGF1R and INSR activates the phosphatidylinositol 3-kinase (PI3K)/threonine protein kinase (Akt), mitogen activated protein kinase (MAP), the mammalian target of rapamycin (mTOR) pathways and regulates cell division and survival [12]. It has been reported that IGF2 mediated activation of the insulin pathway is a well-established mediator of muscle differentiation and proliferation [7]. PI3Ks, a family of lipid kinases that are activated by growth factors, hormones, and cytokines, play key roles in cell growth, proliferation, and differentiation [13]. PI3Ks can be divided into three classes (PIK3Cα, PIK3Cβ and PIK3Cγ) and are regulated by a variety of mainly negative-feedback factors [14]. It has been reported that PI3K3Cβ, as an intermediate factor, links insulin to PI3K signal transduction by regulating insulin receptor trafficking in mice [14]. To date, the molecular regulatory mechanism of IGFs-PI3K-Akt remains unclear in fibroblasts. The aim of this study was to elucidate the role of IGF2 in fibroblast proliferation from yak species. The expression levels of yak IGF2 were regulated by a recombinant over-expression vector and small interfering RNA (siRNA) silencing to demonstrate the association between the IGFs-PI3K-Akt signaling pathways in cell proliferation. Additionally, these results provide evidence supporting the functional role of IGF2 in improving the production performance of livestock of yak.

## 2. Materials and Methods 

### 2.1. Sample Collection and Cell Culture 

Fresh tissues (liver, kidney, spleen, lung, testis, ovary and muscle) from five randomly selected adult yaks (aged 4 years old) were obtained immediately after slaughter from a slaughterhouse in Tianzhu County (Wuwei City, Gansu province, China). The yak tissue samples were immediately stored at −80 °C. This study was supervised by the Animal Care Commission of College of the Veterinary Medicine, Gansu Agriculture University, China, with ethical code GSAU-AEW-2015-0008.

Fibroblasts cultured from a yak fetus were preserved in our lab [15]. After purification, the fibroblasts were maintained in a humidified atmosphere of 5% CO_2_ at 37 °C in Dulbecco’s Modified Eagle Medium (DMEM, Gibco, Grand Island, NY, USA) containing 10% fetal bovine serum (FBS, Gibco), 2 mM l-glutamine (Sigma-Aldrich, St. Louis, MO, USA), 100 U/mL of penicillin, and 100 μg/mL of streptomycin.

### 2.2. Plasmid Construction, Cloning and Transfection

Total RNA was isolated from the yak fetal fibroblasts using TRIzol reagent (Invitrogen, Carlsbad, CA, USA), following the manufacturer’s protocol. Complementary DNA (cDNA) was synthesized using a FastQuant RT kit (TianGen, Beijing, China), according to the manufacturer’s instructions. The cDNA of the 540 bp fragment of *IGF2* was amplified via polymerase chain reaction PCR (Bio-Rad, Hercules, CA, USA) using Premix Taq DNA Polymerase (TaKaRa, Otsu, Japan) and cloned into the pMD18 vector (System Biosciences, Palo Alto, CA, USA). The flanking ScaI and BamHI restriction sites were created, and the DNA fragment was cloned into pIRES2-EGFP (enhanced green fluorescent protein, EGFP) vector (Clontech, Fremont, CA, USA). The primer sequences (Table S1), used for cloning the full-length *IGF2*, were designed using Primer Premier 5.0 (PREMIER Biosoft, Palo Alto, CA, USA). The recombinant plasmids were produced and transfected according to the manufacturer’s protocol. The over-expression plasmids of *IGF2* were transfected into yak fetal fibroblasts using Lipofectamine 2000 (Thermo Fisher Scientific, Carlsbad, CA, USA) for 0, 12, 24, 48 and 72 h. The siRNA targeting yak *IGF2* was designed and synthesized by Guangzhou RiboBio Co., Ltd. (Guangzhou, China) (Table S1). The siRNA against *IGF2* (100 nM) was transfected into yak fetal fibroblasts cells using Lipofectamine 2000 for 0, 12, 24, 48 and 72 h.

### 2.3. Quantitative Real-Time Polymerase Chain Reaction Assays

Total RNA from transfected cells (overexpression, knock-down and control groups) was extracted using Trizol reagent (Invitrogen). Complementary DNA was synthesized as previously described. The expression levels of related genes in the INS (*IGF1*, *IGF2*, *IGF1R*, *IGF2R* and *IRS1*) and PI3K/Akt (*Akt1* and *PIK3CG*) pathways were detected by quantitative real-time PCR (RT-PCR). RT-PCR was performed using 2 μL of cDNA in a 25 μL reaction volume on an ABI7300 real-time system (ABI Systems, Foster City, CA, USA). SYBR Premix Ex TaqTM II and specific primers were used in each reaction. The primers are shown in Table S1. The expression of the housekeeping gene glyceraldehyde-3-phosphate dehydrogenase (*GAPDH*) was used as an intra-tissue control. The ABI7300 PCR system was used for a two-step standard procedure for amplifying the samples. A denaturation step ran for one cycle at 95 °C for 30 s. The annealing step was run for 40 cycles at 95 °C for 5 s and 60 °C for 31 s. All PCR reactions were performed in triplicate. The results were calculated using the 2^−△△CT^ method [16].

### 2.4. Immunohistochemical Staining and Immunofluorescence Assay

Immunohistochemical staining of yak anti-IGF2 (Bioss, Beijing, China) used a standard avidin-biotin-peroxidase complex method (ABC Staining System, SABC, BOSTER, Wuhan, China). Yak testis samples were fixed in 4% paraformaldehyde. Immunohistochemical staining was carried out as described previously [17]. The transfected cells from the over-expression group were cultured for 12, 24, 48 and 72 h. Transfection efficiency of over-expression vector was observed by fluorescence microscopy (Olympus, Tokyo, Japan). After 48 h, the cells were fixed with 4% paraformaldehyde (pH 7.4) for 30 min. The fixed cells were incubated at 4 °C overnight with monoclonal rabbit anti-IGF2 antibody (1:100, Bioss), and then incubated at 37 °C for 2 h with secondary antibodies conjugated to Texas Red (1:400; PE, Abcam, Cambridge, MA, USA) in the dark. Cell nuclei were stained with 4,6-diamidino-2-phenylindole hydrochloride (DAPI, Sigma–Aldrich). All immunostaining assays were performed at least in triplicate. The images were captured using an Olympus fluorescence microscope (Olympus).

### 2.5. Western Blot Analysis

The total protein was extracted from the yak tissues and transfected cells using lysis buffer (Solarbio, Beijing, China), and then the concentration was determined by a BCA protein assay kit (BOSTER). 100 μg samples were applied to a sodium dodecylsulfate polyacrylamide gel (SDS-PAGE) for Western blot analysis. The blots were electrotransferred onto a polyvinylidene fluoride (PVDF) membrane (Millipore CAT, Billerica, MA, USA), and blocking by Tris-HCl buffer containing 5% (*w/v*) non-fat milk for 2 h in room temperature. The membranes were then incubated overnight at 4 °C with primary antibodies: rabbit monoclonal anti-IGF1 antibody (1:1000; Bioss), anti-IGF2 antibody (1:500), rabbit monoclonal anti-IGF1R antibody (Bioss), rabbit monoclonal anti-IGF2R antibody (1:500; Bioss), anti-Akt1 antibody (1:500; Abcam), anti-PI3KCG antibody (1:500; Abcam), and anti-β-ACTIN antibody (1:200; Bioss). The subsequent procedures were carried out as described previously [17]. All immunoblot assays were performed at least in triplicate. Optical densities of the bands were quantified and scanned using Image-Pro Plus 6.0 (Media Cybernetics Co., Rockville, MD, USA). 

### 2.6. Cell Cycle and Cell Proliferation Assays

Transfected cells were collected using 0.01% trypsin (Gibco) and fixed with 75% ethyl alcohol for 1 h. The fixed cells were incubated at room temperature for 30 min with 1 mg/mL propidium iodide (Beyotime, Najing, China). A flow array cytometers (FACS, BD Biosciences, Franklin Lakes, NJ, USA) was used for analyzing the cell cycle [18,19]. Proliferation assays were performed using a cell counting Kit-8 (CCK-8 kit, Dojindo, Kumamoto, Japan), according to the manufacturer’s protocol [18]. Briefly, yak fetal fibroblasts cells were plated in 96-well plates (Costar, Corning, NY, USA), in triplicate, at approximately 1 × 10^6^ cells/well and cultured for 0, 12, 24, 48 and 72 h. Subsequently, the cells were co-cultured with 10 μL CCK-8 solution for 3 h and the number of living cells per well were measured by absorbance (450 nm) of reduced water-soluble tetrazolium salt (WST) at the indicated time points. All assays were repeated at least 3 times.

### 2.7. Statistical Analysis

Statistical analyses were performed using SPSS version 21.0 (SPSS Inc., Chicago, IL, USA). The data were expressed as the mean ± standard deviation (SD) otherwise indicated. Data were analyzed by the Student’s *t* test (between two groups) or one-way ANOVA analysis (within multiple groups). The statistical graphs were drawn by using Prism version 5.0 (GraphPad software Inc., La Jolla, CA, USA). *P* value less than 0.05 was statistically significant. 

## 3. Results

### 3.1. Expression and Location Analyses of IGF2 in Yak Tissues

**Figure 1:**
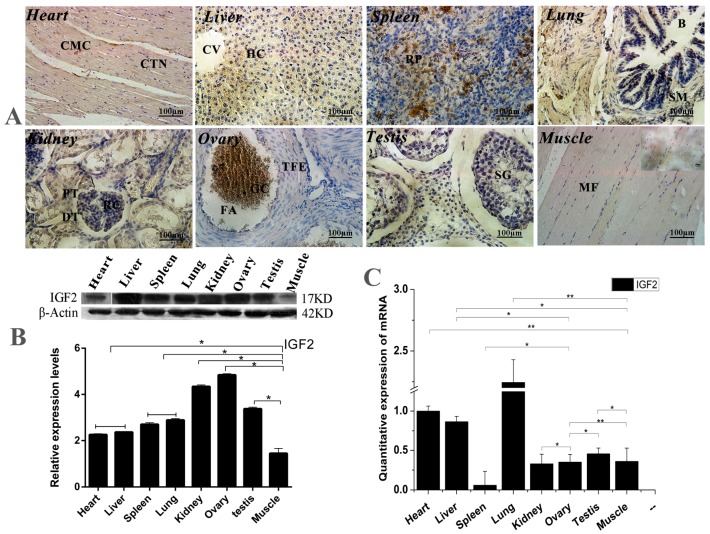
The expression and location analysis of IGF2 in yak tissues. (**A**) Intracellular location analysis of insulin-like growth factor 2 (IGF2) protein in eight different organs from yak (400×). CMC: Cardiomyocytes; CTN: Connective tissue cell nucleus; CV: Central vein; HC: Hepatocyte; RP: Red marrow; B: Bronchioles; SM: Smooth muscle; PT: Proximal convoluted tubule; DT: Distal convoluted tubule; RC: Glomerular; TFE: Theca of follicle of von Baer; FA: Follicular cavity; SG: Stratum granulosum; GC: Granulosa cells; MF: Muscle fiber. (**B**): Western blot analysis of IGF2 protein in eight different organs from yak. (**C**) The quantitative expression level of *IGF2* mRNA detected in yak tissues. Data are presented as the mean ± standard deviation (SD) of five independent experiments for each sample. * *p <* 0.05, ** *p <* 0.01.

The expression and location analyses of yak IGF2 were carried out in eight different organs from yak, and the mRNA and protein of yak IGF2 were found to be expressed differentially in the tissues (Figure 1). Mostly, IGF2 proteins were mainly found in cytoplasm (Figure 1A), although the positive expression level was differed among different tissue. The expression of IGF2 was shown to be weakly-reactive in skeletal muscle and heart muscle. A few cells with nuclear immunostaining were observed in bronchiolar epithelial tissue, possibly infiltrating cells. A weakly immune-positive reaction was observed in bronchiolar smooth muscle cells (Reissesen membrane). The majority of pneumocytes were shown to be negative, while the alveolar lining cells displayed a weak/moderate cytoplasmic immunoreaction in the lung parenchyma. The white pulp cells were mainly negative, whereas those in the red pulp showed moderate or strong cytoplasmic immunostaining in the spleen. In the kidneys, the renal corpuscles were usually negative or weakly positive for IGF2 protein, but the tubular epithelium was strongly immunopositive. Hepatocytes showed a moderate cytoplasmic staining without a nuclear immunoreaction. In the ovaries, granulosa cells frequently displayed a moderate cytoplasmic immunostaining, while theca of follicle showed a weak cytoplasmic staining. The spermatogonia, spermatocytes, spermatids and Sertoli cells in the testis were not immunostained for IGF2 protein. Conversely, Leydig cells displayed moderate/strong cytoplasmic positivity. The highest expression level of IGF2 protein was shown in the lung, while the lowest expression level of IGF2 protein was shown in muscle tissue (Figure 1B). However, the highest expression level of *IGF2* mRNA was also shown in the lung, and the lowest expression level of *IGF2* mRNA was seen in the spleen (Figure 1C). These results suggested that the expression of IGF2 in muscle was lower than that of other tissues, which could be affected by the yak growth circumstance.

### 3.2. Transfected Effect of Over-Expression and Knockdown of Yak IGF2 

**Figure 2:**
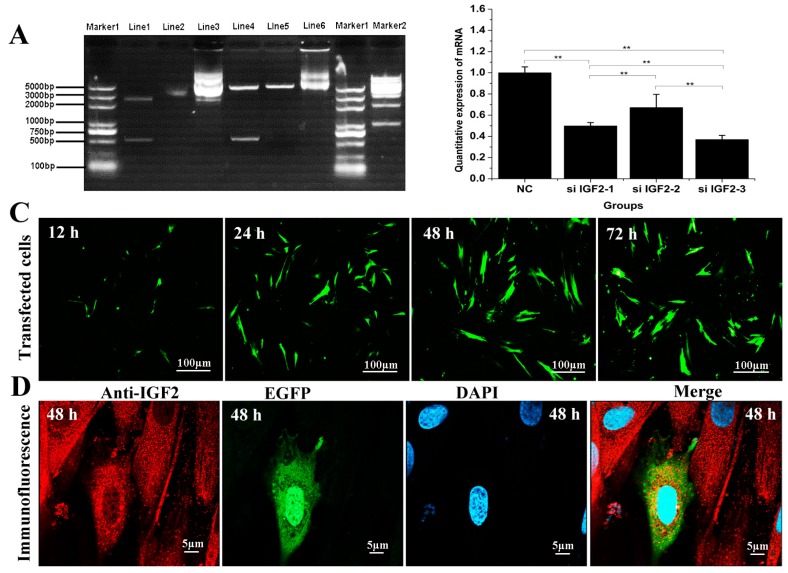
Effect of over-expression and knockdown of IGF2 in yak fibroblasts. (**A**) Double restriction enzyme was carried out to identify the recombinant vector, and a fragment of almost 500 bp was detected, in accordance with the coding sequence (CDS) of yak *IGF2*. Marker 1 and 2: DNA Marker. Line1: double restriction enzymes of pUC57-*IGF2* plasmid. Line2: pUC57-*IGF2* plasmid. Line3: pIRES2-EGFP-*IGF2* plasmid. EGFP: enhanced green fluorescent protein. Line4: double restriction enzymes of pIRES2-EGFP-*IGF2*. Line5: double restriction enzymes of pIRES2-EGFP. Line6: pIRES2-EGFP plasmid. (**B**) The knockdown efficiency was analyzed and the si-*IGF2*-3 was the most efficient sequence. (**C**) Transfection effect of over-expression vector on *IGF2* with different time points, and the transfected cells were gradually by the time. (**D**) Cellular localization of IGF2 in yak fibroblasts. The expression of IGF2 was in the cytoplasm and the expression level increased by the time. Data are presented as the mean ± SD of three independent experiments for each small interfering RNA (siRNA). * *p <* 0.05, ** *p <* 0.01.

To confirm the effect of *IGF2* over-expression or knockdown on yak fibroblast cells, we designed a series of experiments including PCR and cell immunofluorescence (Figure 2). Double restriction enzyme (SacI and BamHI) was carried out to identify the recombinant vector, and a fragment of almost 500 base pair (bp) was detected. This size was in accordance with the coding sequence (CDS) of yak *IGF2* (Figure 2A). The efficiency of target gene knockdown using siRNA was also analyzed, and the results suggested si-*IGF2*-3 sequence was the most efficient candidate and it has shown up to 63% of knockdown efficiency (Figure 2B). EGFP protein was tagged together with *IGF2* into over-expression vector and it could be observed via fluorescence microscope. However, there was no obvious fluorescence signal in siRNA sequence. Therefore, the transfection effect and cellular localization were observed only in the over-expression vector group. The results revealed that the transfected cells gradually increased with prolonged time (Figure 2C). The expression of IGF2 was in the cytoplasm where the expression level also increased by the time (Figure 2D). 

### 3.3. The Effect of IGF2 Over-Expression on IGF mRNA and Protein 

**Figure 3:**
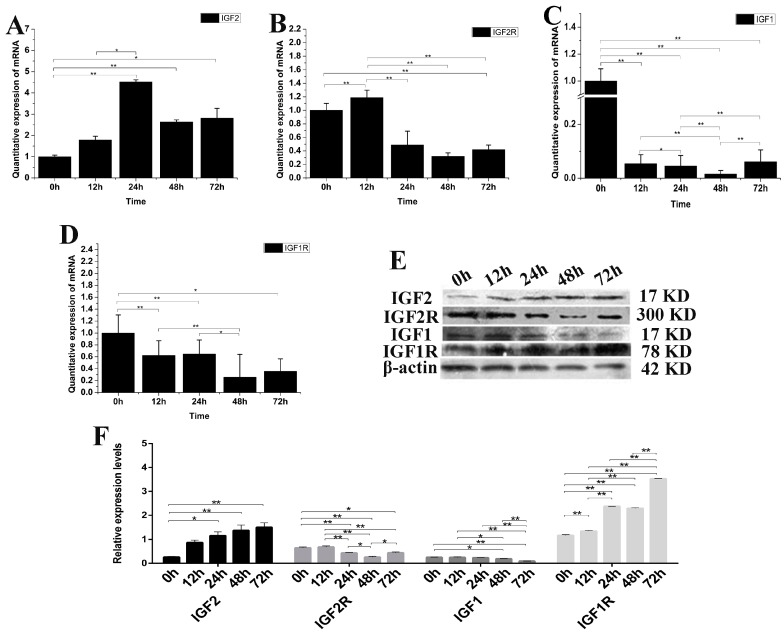
The changes of IGF2 overexpression on IGF mRNA and protein. (**A**–**D**) The expression levels of *IGF2*, *IGF2R*, *IGF1* and *IGF1R*. The expression of *IGF2* was up-regulated, resulting in down-regulation of *IGF2R*, *IGF1* and *IGF1R* in fibroblasts. (**E**) The expression of IGF proteins were analyzed by western blot. (**F**) The relative expression levels were calculated according to the relative ratio of optical densities of IGFs against the values of β-ACTIN. The expression of IGF2 protein was up-regulated, causing the expression of IGF2R and IGF1 to be down-regulated in fibroblasts. Meanwhile the expression of IGF1R was significantly up-regulated. Data were presented as the mean ± SD of three independent experiments for each time point. * *p <* 0.05, ** *p <* 0.01.

*IGF2* can mediate muscle cell differentiation and proliferation via regulation of the IGF pathway. The expression of *IGF1*, *IGF2*, *IGF1R* and *IGF2R* mRNA and protein were analyzed after over-expression of the *IGF2* gene in fibroblasts (Figure 3). The expression of *IGF2* mRNA and protein were significantly up-regulated compared to the control (*p <* 0.01). The highest expression level of *IGF2* mRNA was detected at 24 h after transfection (Figure 3A), while the highest expression level of IGF2 protein was detected at 72 h after transfection. The expression of *IGF2R*, *IGF1* and *IGF1R* mRNA were down-regulated (Figure 3B–D), but particularly *IGF1* which was significantly different compared to that of *IGF1R*, *IGF2R* and the control (*p* < 0.01). The expression of IGF2R and IGF1 proteins were down-regulated, but without any obvious difference compared to that of IGF2. However, the expression of IGF1R protein was up-regulated compared to the control (*p* < 0.01), and was significantly different compared to that of IGF1, IGF1R and IGF2 (Figure 3E,F). These results suggested that the *IGF2* can mediate the genes expression levels in IGF pathway.

### 3.4. The Effect of IGF2-Knockdown on IGF mRNA and Protein 

**Figure 4:**
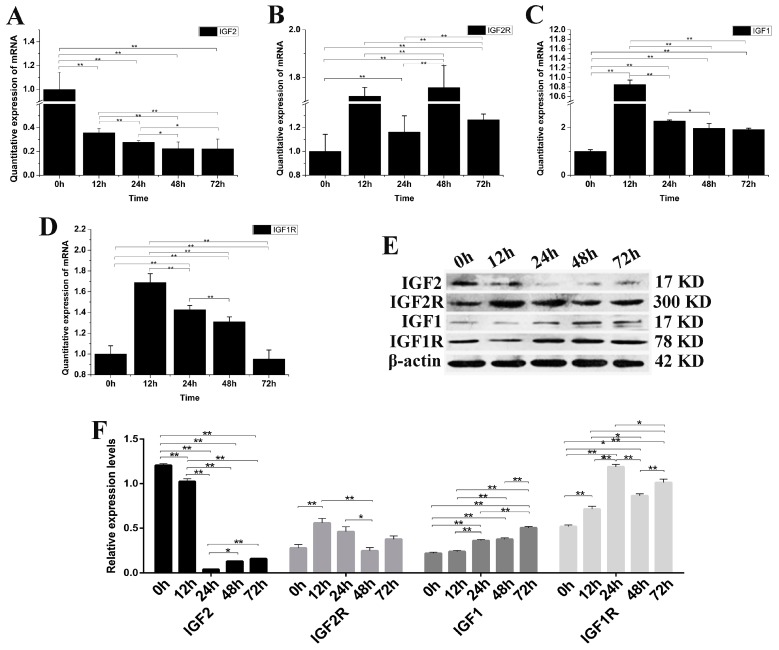
The changes of IGFs mRNA and protein after knockdown yak IGF2. (**A**–**D**) The expression levels of *IGF2*, *IGF2R*, *IGF1* and *IGF1R*. (**E**) The expression of IGF proteins were analyzed by western blot. (**F**) The relative expression levels were calculated according to the relative ratio of optical densities of IGFs against the values of β-actin. The expression of *IGF2* mRNA and protein were down-regulated, resulting in the expression of IGF1, IGF1R and IGF2R being up-regulated in yak fibroblasts. All data were presented as the mean ± SD of three independent experiments for each time point. * *p <* 0.05, ** *p <* 0.01.

*IGF2* gene expression was knocked down by siRNA-*IGF2*-3 to verify once more the role of IGF2 in yak fibroblast (Figure 4). The expression of *IGF2* mRNA and protein were significantly down-regulated compared to the control group (*p <* 0.01) (Figure 4A). The expression of *IGF2R*, *IGF1* and *IGF1R* mRNA were up-regulated. *IGF1* was significantly different at 12 h after transfection, compared to that of *IGF1R* and *IGF2R* (Figure 4B–D). The expression of IGF2R and IGF1 proteins were up-regulated, but without obvious differences compared to that of IGF2. The expression of IGF1, IGF1R and IGF2R proteins were up-regulated compared to the control (*p* < 0.01) and were significantly different than the expression of IGF2 (Figure 4E,F). These results suggested that the expression of IGF1R was significantly regulated via the expression of *IGF2*.

### 3.5. The Effect of IGF2 Over-Expression on the PI3K-Akt Signaling Pathway

**Figure 5:**
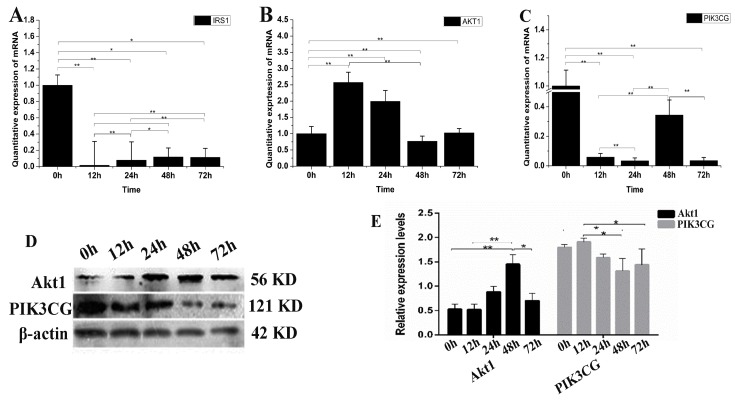
The possible effect of *IGF2* over-expression on the PI3K-Akt signaling pathway. (**A**–**C**) The expression levels of *IRS1*, *Akt1* and *PIK3CG*. (**D**) The expression of Akt1 and PIK3CG proteins were analyzed by western blot. (**E**) The relative expression levels were calculated according to the relative ratio of optical densities of Akt1 and PIK3CG against the values of β-actin. The expression of *IGF2* was up-regulated, inhibiting the expression of IRS1 and PIK3CG. Meanwhile, the expression of Akt1 was up-regulated. The expression level of Akt1 protein was increased as differentiation progressed, while the reduction of PIK3CG protein expression was much more pronounced. All data were presented as the mean ± SD of three independent experiments for each time point. * *p <* 0.05, ** *p <* 0.01.

IGF promotes cell differentiation via the PI3K-Akt pathway. Therefore, we examined the possible changes of *IGF2* over-expression on PI3K-Akt signaling (Figure 5). The expression level of *IRS1* mRNA was down-regulated in yak fibroblasts over-expressing *IGF2* (Figure 5A). The expression levels of *Akt1* at 12 and 24 h were up-regulated approximately several-folds higher than the control (Figure 5B). The expression level of *PIK3CG* (Figure 5C) was significantly down-regulated at each time point, compared to the control (0 h). The expression level of Akt1 protein increased as differentiation progressed under conditions of *IGF2* over-expression, and remained extremely high over time, especially at 48 h. In contrast, the reduction of PIK3CG protein expression was much more pronounced (Figure 5D,E). The results suggested that *IGF2* could participate and regulate PI3K-Akt pathway.

### 3.6. The Effect of IGF2 Knockdown on PI3K-Akt Signaling Pathway

**Figure 6:**
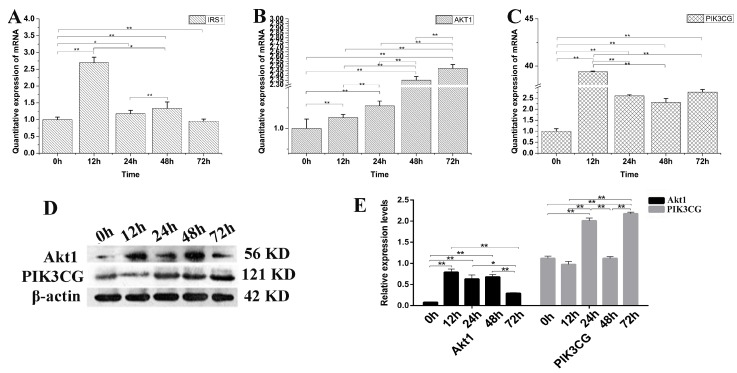
The possible effect of *IGF2* knockdown on PI3K-Akt signaling pathway. (**A**–**C**) The expression levels of IRS1, Akt1 and PIK3CG. (**D**) The expression of Akt1 and PIK3CG proteins were analyzed by western blot. (**E**) The relative expression levels were calculated according to the relative ratio of optical densities of Akt1 and PIK3CG against the values of β-actin. The expression of IGF2 was down-regulated, promoting the expression of IRS1, Akt1 and PIK3CG. The expression level of Akt1 and PIK3CG proteins were increased as differentiation progressed. All data were presented as the mean ± SD of three independent experiments for each time point. * *p* < 0.05, ** *p <* 0.01.

To verify the decreased PI3K-Akt expression is *IGF2* dependent or not, the yak fibroblasts were transfected with *IGF2* siRNA and the expression levels of related genes and proteins in the PI3K-Akt signaling pathway were examined (Figure 6). The expression level of *IRS1* mRNA was significantly higher at 12 h than that of other time points (Figure 6A). As expected, the expression levels of *Akt1* (Figure 6B) and *PIK3CG* (Figure 6C) were up-regulated several-fold, compared to the control (0 h) in *IGF2* knockdown cells. Interestingly, the expression of PIK3CG and Akt1 proteins (Figure 6D,E) increased rapidly and significantly at each point, compared to the control (0 h). Meanwhile, the levels of PIK3CG were significantly higher at 24 h and 72 h (*p <* 0.01) after *IGF2* knockdown. The results suggested that the PI3K-Akt activation was dependent on the expression of *IGF2*.

### 3.7. Cell Cycle and Proliferation after IGF2 Over-Expression or Knockdown 

**Figure 7:**
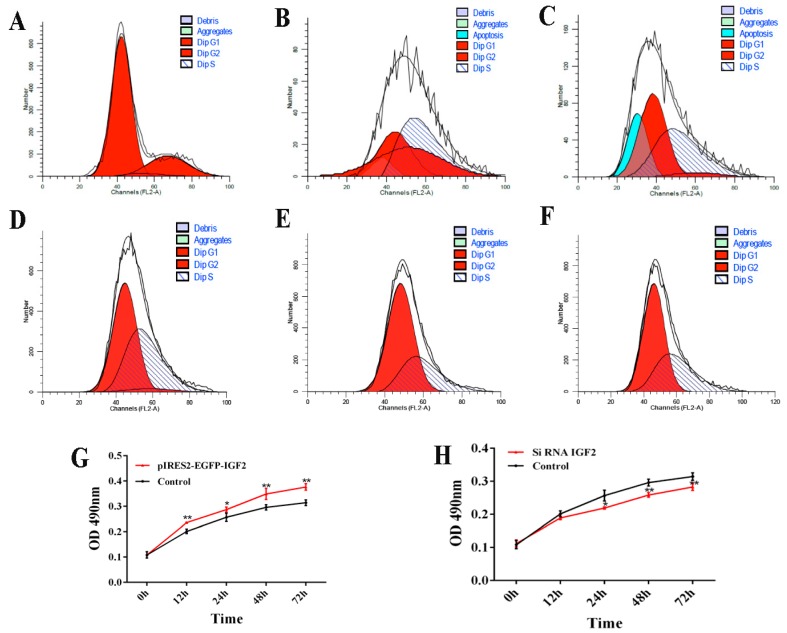
Cell cycle and proliferation after *IGF2* over-expression or knockdown. (**A**–**F**) The cell cycle of yak fetal fibroblasts was examined by flow cytometry after IGF2 over-expression or knockdown. (**A**) control. (**B**) *IGF2* over-expression transfected for 24 h. (**C**) IGF2 over-expression transfected for 48 h. (**D**) control siRNA. (**E**) *IGF2* siRNA transfected for 24 h. (**F**) *IGF2* siRNA transfected for 48 h. Dip: diploid. (**G**) and (**H**) The cell proliferation of yak fetal fibroblasts was examined after *IGF2* over-expression or knockdown by cell counting Kit-8 (CCK-8) assays. The results revealed the over-expression of *IGF2* can enhance DNA synthesis phase and cause proliferation, while the knockdown of *IGF2* can decrease DNA synthesis and inhibit proliferation.

The cell cycle and proliferation of yak fetal fibroblasts were examined by flow cytometry and CCK-8 after over-expression or knockdown *IGF2* (Figure 7). When *IGF2* was over-expressed, the percentage of cells in the G1 phase of the cell cycle was 74.41% (0 h), 26.77% (24 h) and 45.16% (48 h) (Figure 7A–C). Compared to the control (0 h), the percentage of cells in the G1 phase was significantly reduced. The percentage of cells in the S phase was 3.72% (0 h), 38.06% (24 h) and 48.90% (48 h). The proportion of cells in the S phase gradually increased by the transfection time. When *IGF2* was inhibited, the percentage of cells in the G1 phase was significantly increased, compared with the control, those in the S phase were decreased, compared with the control. However, the G1 and S phases had no significant differences after transfection for 24 h and 48 h (Figure 7D–F). As indicated in the CCK-8 assay (Figure 7G,H), the fibroblast proliferation trend in the over-expression group was significantly higher than that of the control (Figure 7G). Fibroblasts transfected with *IGF2* siRNA showed significant inhibition of cell proliferation (Figure 7H). These results revealed that the regulated expression of *IGF2* can promotes fibroblast proliferation.

## 4. Discussion

Increasingly, studies have confirmed that the IGFs play an important role in regulating muscle proliferation and differentiation [20], and previous results suggest that IGFs exert their effects on different cell types through different receptors, such as IGF1R and INSR [21]. However, the roles of IGF2 and IGF2R are less clear. The demonstration of induction of *IGFs* mRNAs in muscle tissues in response to a growth stimulus prompted examination of IGFs gene expression during muscle differentiation [22]. The results of immunohistochemically staining showed that IGF2 was located mainly in myocytes of the different tissues. The expression levels of *IGF2* mRNA and protein were differentially expressed in different tissues. The highest expression levels of *IGF2* mRNA were observed in the lung and the highest expression levels of IGF2 protein were found in the ovaries and kidneys. It has been shown that, in mice, the highest expression of IGF2 was observed in the liver and kidney and changed with growth and development. However, the results were contradictory to our study. In order to survive and reproduce in hypoxic and high altitude environments, the lungs and ovaries of yaks are different than that of other animals [23]. Moreover, *IGF2* is an imprinted gene, expressed in a monoallelic or biallelic manner, depending on parental legacy [24]. This suggests that IGF2 plays an important role in growth and differentiation in animal development. These are possible reasons for the production performance, body size and mass of yak being lower than that of cattle.

In the present study, we showed that the changes of *IGF2* gene expressions can affect the expression levels of *IGF2* mRNA and proteins significantly, compared to the control group. The results indicated that whether the expression of *IGF2* was up-regulated or down-regulated, the expression of IGF1R was significantly different compared to that of IGF1 and IGF2R. It was suggested that *IGF2*-mediated activation of the insulin pathway has a directly effect on IGF1R in yak fibroblasts. It was also demonstrated that the direct integrin binding to IGF2 through the C-domain is required for IGF1R in induced proliferation of CHO (Chinese hamster ovary) cells [25]. 

The biological actions of IGFs are mediated through IGF1R in zebrafish, and signaling through IGF1R is transduced primarily by the PI3K-Akt [26]. Subsequently, the effect of *IGF2* over-expression or knockdown on PI3K-Akt signaling was analyzed in yak fibroblasts. We found that the expression of *PI3KCG* mRNA and protein were the most conspicuous target molecule in the PI3K-Akt pathway, which appears in a dynamic variation with the expression of IGF1R. PI3KCG, as one member of the subclass of PI3Ks [27], is an important mediator for the regulation of cell proliferation via PI3K-Akt pathways [28]. The results indicated that the *IGF2* gene had negative regulatory effect on *PIK3CG* through binding with *IGF1R*. Furthermore, the cell cycle was prolonged after regulating the expression of *IGF2*, particularly in the G1 and S phases for 12 h in fibroblasts. The CCK-8 assay results also suggested that the proliferation trend of *IGF2* over-expression and knockdown for 12 h were most obvious compared against other time points. It was reported that IGF1 withdrawal in the mid-G1 phase impaired the association of PI3K with IGF1R and suppressed DNA synthesis, similarly to addition of a PI3K inhibitor [29]. The S phase of cell cycle was obviously affected after regulating the expression level of *IGF2*, causing either fibroblast proliferation promotion or inhibition. The conclusion is that continuous stimulation with IGFs is required for IGF-induced cell proliferation [30,31], and this role of IGFs as cell cycle progression factors is distinctive from those of many other growth factors [29].

## Supplementary Materials

The following are available online at http://www.mdpi.com/2073-4425/9/3/169/s1, Table S1: Primers information of PCR and qPCR.
