# Supplementary material for: Yak IGF2 Promotes Fibroblast Proliferation Via Suppression of IGF1R and PI3KCG Expression
Source: Genes (Basel). 2018 Mar 20;9(3):169. doi: 10.3390/genes9030169 (PMC5867890; doi:10.3390/genes9030169)
Supplement: Supplementary file 1 [file genes-09-00169-s001.pdf]

Supplementary Table S1

Table S1 Primers information of PCR and qPCR

Primer name	Primer sequence	Products	Genebank No.
AKT1-F	GGGAAGGTGATCCTGGTGAA	115	XM_005911925.2
AKT1-R	GGTTCTCTGTGAGCGTGTGG		
IGF1-F	CATCACATCCTCCTCGCATC	146	KJ489376
IGF1-R	TGAAATAAAAGCCCCTGTCTCC		
IGF1R-F	CCAGAGCAAAGGGGACATAAA	87	KF303539.1
IGF1R-R	GTGGTGGTGGAGGTGAAGTG		
IGF2-F	CTGGTGCTTCTTGCCTTCTTG	259	KF682139.1
IGF2-R	CAGACACATCCCTCTCGGACTT		
IGF2R-F	GCACGGCAACCTGTATAACCT	235	XM_014479665.1
IGF2R-R	CATCTTCAGCACCCCATTCTC		
IRS1-F	TGGATGCAGGTGGACGA	175	XM_005890133.2
IRS1-R	TGGGTGGAGGGTTGTTGAG		
PIK3CG-F	TCAATGTCCATCTCCATTCTCCT	138	XM_005901435.2
PIK3CG-R	GATTGCCTCCAGTTGCTTCC		
GAPDH-F	CAACGGCACAGTCAAGGCA	232	EU195062.1
GAPDH-R	CATCACAAACATGGGGGCA		
Yak IGF2	CGAGCTCGCCACCATGGGGATCACAGCAGGAAA<br>CGGGATCCCTAATCGCTGGATGCCTTGG	540	KF682139.1
siRNA-yak IGF2	UCCAAGGCAUCCAGCGAUUdTdT<br>dTdTCAGGUUCCGUAGGUCGCUAA		KF682139.1
